# Assessment of Packaged Foods and Beverages Carrying Nutrition Marketing against Canada’s Food Guide Recommendations

**DOI:** 10.3390/nu11020411

**Published:** 2019-02-15

**Authors:** Beatriz Franco-Arellano, Min Ah Kim, Stefanie Vandevijvere, Jodi T. Bernstein, Marie-Ève Labonté, Christine Mulligan, Mary R. L’Abbé

**Affiliations:** 1 Department of Nutritional Sciences, Faculty of Medicine, University of Toronto, Toronto, ON M5S 1A8, Canada; beatriz.francoarellano@mail.utoronto.ca (B.F.-A.); minahk.kim@mail.utoronto.ca (M.A.K.); jodi.bernstein@mail.utoronto.ca (J.T.B.); Marie-Eve.Labonte@fsaa.ulaval.ca (M.-È.L.); christine.mulligan@mail.utoronto.ca (C.M.); 2 Dalla Lana School of Public Health, University of Toronto, Toronto, ON M5S 1A1, Canada; 3 Department of Epidemiology and Biostatistics, School of Population Health, University of Auckland, Auckland 1010, New Zealand; s.vandevijvere@auckland.ac.nz; 4 School of Nutrition & Institute of Nutrition and Functional Foods, Laval University, Québec, QC G1V 0A6, Canada

**Keywords:** dietary guidelines, Canada’s food guide, INFORMAS, health Canada surveillance tool, nutrition marketing, nutrient and health claims, food supply

## Abstract

Canadians’ food purchases consist largely of packaged processed and ultra-processed products, which typically fall outside the “core” foods recommended by Canada’s Food Guide (CFG). Almost half of packaged products in Canada carry nutrition marketing (i.e., nutrient content and health claims). This study assessed whether packaged foods carrying nutrition marketing align with recommendations outlined in the 2007 CFG. Label data (*n* = 9376) were extracted from the 2013 Food Label Information Program (FLIP). Label components (including nutrition marketing) were classified using the International Network for Food and Obesity/NCDs Research, Monitoring and Action Support (INFORMAS) labelling taxonomy. The Health Canada Surveillance Tool (HCST) was used to assess the alignment of products to CFG. Each food or beverage was classified into one of five groups (i.e., Tier 1, Tier 2, Tier 3, Tier 4, “Others”). Products in Tier 1, 2 or water were considered “in line with CFG”. Most products in the analyzed sample were classified as Tier 2 (35%) and Tier 3 (27%). Although foods with nutrition marketing were significantly more likely to align to CFG recommendations (*p* < 0.001), many products not “in line with CFG” still carried nutrition marketing. This study provides important baseline data that could be used upon the implementation of the new CFG.

## 1. Introduction

The increase in obesity and diet-related non-communicable diseases (NCDs) is a concern and action needs to be taken to reduce this global health burden [1]. More worrisome is the fact that diet-related mortality is not only increasing but appearing earlier in life, affecting both developed and developing countries [1,2,3]. Therefore, adherence to a healthy diet remains critical to prevent obesity and NCDs, as well as all forms of malnutrition [4]. 

Dietary guidelines aim to promote healthy diets and lifestyles through country-specific and science-based agricultural, food, health and nutrition recommendations [5]. Many countries have issued and/or updated national dietary guidelines, and reviews of these have been published worldwide [6,7]. Adherence to national dietary guidelines has been found to be associated with reductions in diet-related risk factors and obesity [8,9,10,11,12,13]. Since first released in 1942, Canada’s Food Guide (CFG) has communicated dietary guidance to Canadians [14]. However, the diets of Canadians have been found to be only partially aligned with CFG’s recommendations [15]. Public consultations launched in 2016 and 2017 to revise CFG (issued in 2007 and upgraded in 2019) have highlighted the need to create greater awareness of the nutritional quality of foods through strategies such as providing simple and accurate nutrition labelling to consumers, developing schemes that facilitate decision-making when purchasing foods, assessing the impact of ultra-processed foods on health, and limiting intakes of processed or prepared foods and beverages with high sodium, sugars or saturated fat content, among others [16,17,18]. As such, dietary guidelines can be used as policy tools to influence not only diet recommendations (e.g., daily fruit and vegetable intake), but also the food environment (e.g., school food programs, stimulating product reformulation by industry) [5,6]. 

The food environment, defined as “the collective physical, economic, policy and sociocultural surroundings, opportunities and conditions that influence people’s food and beverage choices and nutritional status“ [19], has been found to play a critical role in driving diets [20]. For instance, an unhealthy food environment is known to induce unhealthy diets (i.e., diets characterized by low consumption of fruit and vegetables, and high consumption of foods with high contents of sodium, sugars, saturated and trans fats [1]), and energy overconsumption by increasing the availability and affordability of processed and ultra-processed foods, which are foods commonly high in added fat, sugars and/or sodium [21,22]. In Canada, >60% of dietary energy is derived from ultra-processed foods [23], most of which fall outside the “core” foods recommended by CFG [24]. Processed and ultra-processed foods also tend to carry a considerable number of nutrient content and health claims (hereafter referred to as “nutrition marketing”) [25,26,27,28,29], which is a significant driver of consumers’ purchasing choice [30,31]. Canadians consume the majority of their calories from foods prepared at home; however, meals have been found to exceed the recommendations for saturated fat, sugars and sodium [32], which could reflect the rise in use of packaged foods and ready-to-eat meals at home [32,33,34]. Such changes in consumer behavior could be derived from the trade-offs between convenience, time, availability and the nutritional quality of foods consumers often face when making foods choices [34]. 

It has been suggested that attitudes towards healthy eating could be improved if the food environment (including the food supply) facilitated healthier food choices [1,3,35]. Hence, there has been a particular focus on the implementation of policies and programs that influence holistic changes to the food environment, such as strengthening national dietary guidelines along with other targeted policies, like regulating the marketing of foods with low-nutritional quality [5]. These cost-effective policies and programs can be useful for reducing obesity and other NCDs risk factors [36,37,38,39,40,41,42,43,44]. The CFG (upgraded in 2019) advises consumers to be aware of the use of nutrition marketing on food labels; however, little data assessing the association of foods with nutrition marketing to its predecessor is available. Therefore, considering that Canadians’ food purchases come largely from packaged foods, nutrition marketing is highly prevalent on these foods, and being aware of marketing practices is one key recommendation of the CFG’s newest version, the primary objective of this study was to assess whether packaged foods carrying nutrition marketing are in line with the 2007 Canada’s Food Guide recommendations, as baseline data upon the implementation of the revamped guideline. A secondary objective was to determine the use of other label components (e.g., supplementary nutrition information) that could also provide consumers with other tools to make decisions. 

## 2. Materials and Methods 

Food and beverage products that have been shown to be associated with increased risk of obesity and diet-related NCDs worldwide (e.g., convenience foods or packaged meals, sugar sweetened beverages, refined grains, processed meats), and foods associated with healthier food patterns (e.g., high consumption of fruits, vegetables, nuts and legumes) [3,36,45,46,47,48] (*n* = 9376) were included as part of this convenience sample of products selected from the 2013 Food Label Information Program (FLIP) [49]. This sample reflects foods commonly consumed by Canadians (e.g., energy-dense, high-fat, low–fiber foods [47]).

FLIP 2013 (*n* = 15,342) is a database that contains label information for Canadian packaged foods and non-alcoholic beverages [49]. Briefly, FLIP 2013 data were acquired by examining grocery store shelves and photographing food labels of packaged foods and beverages that were on grocery store shelves from the top four Canadian grocers (Loblaws, Metro, Safeway, and Sobeys), which represented approximately 75% of the Canadian grocery retail market share [49]. The data collection took place between May and September 2013. Information was collected from all products bearing a mandatory Nutrition Facts table (NFt), including all flavour variations of national brands and private labels, but only one package size of each product was collected. Products were excluded from collection if an NFt was not displayed on the label (e.g., breads baked at the store). Products were also not collected if they were seasonal products (e.g., Easter chocolates, Christmas eggnog), natural health products, alcoholic beverages or baby foods. Data were collected using smartphones and uploaded onto the FLIP website, specially designed to store food label information. Trained staff extracted and verified label information such as nutrition information, list of ingredients, brand, container size, universal product code (UPC) and price [25,49].

### 2.1. Classification of Label Components

Label information was classified using the International Network for Food and Obesity/NCDs Research, Monitoring and Action Support (INFORMAS) food labelling taxonomy [44]. INFORMAS is “a global network of public interest organizations and researchers that aims to monitor and benchmark food environments and support public and private sector actions to reduce NCDs and obesity, and their inequalities” [20]. The INFORMAS taxonomy is an internationally standardized methodology for the collection and comparison of nutrition-related marketing on packaged foods and beverages [44]. The purpose of using the INFORMAS taxonomy was two-fold: to allow objective comparison with similar studies that have investigated nutrition marketing in the food supply worldwide [27,29,50,51,52,53], and to minimize bias towards identification of other label components that are not currently mandated in Canada (e.g., supplementary nutrition information and quantitative ingredient declaration). This taxonomy divides nutrition-related labelling on food packages into the following two components: (1) nutrition information and, (2) nutrition and health claims [48]. 

#### 2.1.1. Nutrition Information

- List of ingredients: presence/absence of a list of ingredients and other aspects of the list of ingredients (e.g., whether a quantitative ingredient declaration [QUID] was made). The % symbol was searched for in the list of ingredients to verify products with QUID. Products were not considered as displaying QUID if the quantification referred to: % additives (e.g., “contains 2% or less of each of the following: sodium aluminum phosphate, baking soda, artificial flavors, salt), % origin (e.g., “100% Canadian milk”), % organic (e.g., “100% organic ingredients”).
- Nutrient declarations: presence/absence of the Nutrition Facts table.
- Supplementary nutrition information: presence/absence of interpretive nutrition information, such as traffic light labeling (TLL), health star ratings (HSR), or guideline daily amounts (GDA).

#### 2.1.2. Nutrition and Health Claims (i.e., Nutrition Marketing)

The INFORMAS taxonomy divides claims into 3 major categories [44] which are:

- Nutrition claims: including nutrient content claims, nutrient comparative claims and health-related ingredient claims).
- Health claims: including general health claims, nutrient and other function claims, and reduction of disease risk claims). Logos or heart-shaped symbols (such as the ones used by national heart foundations like the British Heart Foundation or the Australia/New Zealand Heart Foundation Tick) were considered as reduction of disease risk claims because they seem to imply a relationship between the consumption or a product and cardiovascular disease risk [44]. In Canada, a similar symbol was used at the time of data collection (the Heart and Stroke Foundation [HSF] logo) and therefore was classified as such for the propose of this study; however, it is important to note that the logo did not comply with the Canadian regulations for disease risk reduction claims and therefore it could instead be considered as an unregulated general health claim. The HSF logo was discontinued in 2014 (a year after data was collected).
- Other claims: included other health-related claims (e.g., “gluten-free” claims) and environment-related claims (e.g., “organic”), but they were not analyzed in the present study as they are not considered “nutrients”.

Examples of nutrition and health claims as per the INFORMAS taxonomy are shown in Supplementary Table S1. Label components were identified and extracted by two researchers (B.F.-A., M.A.K.) by reviewing photographs of each individual food label included in the current study. An Excel database was created in which label components were coded for each food or beverage and which was later validated for accuracy. If uncertainties about classification arose, such doubts were discussed among researchers and a final classification was agreed upon.

### 2.2. Food Category Classification

The Global Food Monitoring Group (GFMG) system was used to classify the selected sample of foods into food categories, as established by the INFORMAS labelling protocol (see Supplementary Table S2 for details of the GFMG food categories) [48,54]. Food products were classified into the following food categories: beverages; bread and bakery products; cereal and grain products; confectionery; desserts and ice cream and edible ices; eggs; fruits and vegetables (including nuts and legumes); snack foods; processed fish; meat and meat alternatives; sauces, dressings and condiments; and sugar, honey and related products. 

### 2.3. Classification of Products According to Health Canada Surveillance Tool Nutrient Profiling System

The Health Canada Surveillance Tool (HCST) is the first government-based Canadian nutrient profiling system (NP), and was developed to assess Canadians’ adherence to the 2007 Canada’s Food Guide (CFG), in terms of the amount and the nutritional quality of food choices [55]. The HSCT has been validated against the World Health Organization’s (WHO) recommendations of the nutrients/food components that characterize “healthy” and “unhealthy” diets (i.e., content validity), as well as its ability to characterize the nutritional quality of foods in the Canadian context compared to other NP models (i.e., construct/convergent validity) [1,56]. The HCST classifies foods within each of the CFG’s food groups (i.e., Vegetables and Fruits, Grain products, Milk and alternatives, Meat and alternatives) into four tiers, based on their fats, sugars and sodium content (Table 1). A fifth group, “Others”, which consists of foods and beverages that fall outside of the four food groups (i.e., high calorie beverages (≥40 kcal/100 g), low calorie beverages (<40 kcal/100 g), high fat and/or sugar foods, meal replacements, saturated and/or trans fats and oils, supplements, ingredients/seasonings, water and unsaturated fats and oils) was also included. Each food or beverage in FLIP 2013 was classified into one of those five groups (i.e., Tier 1, Tier 2, Tier 3, Tier 4 and “Others”). Algorithms were developed by the research team (M.-È.L, B.F.-A., J.T.B., C.M.) to systematically classify foods and beverages in FLIP 2013 using the HCST Tier system. Foods were considered to be “in line with CFG” if they had been classified as Tier 1, Tier 2 and “other foods and beverages recommended in CFG”, such as water and unsaturated fats and oils. Unsaturated fats and oils were not found in this sample, otherwise, such products would also have been considered to be “in line with CFG“. The rest of the foods and beverages were considered to be “not in line with CFG” (Table 1).

### 2.4. Data Analysis

The proportion (%) of different label components in this sample of Canadian foods was calculated overall and by food category. The proportion (%) of products in each tier (i.e., Tier 1, 2, 3, 4, and “Others”) was calculated overall, by food category and by the types of nutrition and health claims. The proportion (%) of foods and beverages that were considered “in line with CFG” (i.e., Tiers 1 and 2, and “other foods and beverages recommended in CFG”, such as water and unsaturated fats and oils) and those considered “not in line with CFG” was also calculated for products with and without each type of claim. Binomial logistic regression models (one per type of claim) were used to examine the association between the presence and nature of nutrition marketing (i.e., type of claim) and the adherence of products bearing such claims to the CFG (i.e., “in line with CFG”) compared to those “in line with CFG” but without claims. Analyses were conducted using the statistical software package R. A *p* value of less than 0.05 was deemed statistically significant.

## 3. Results

### 3.1. Label Components

Table 2 provides an overview of the different label components displayed on Canadian foods, as determined by the INFORMAS food labelling taxonomy. Since the Nutrition Facts table and Ingredients List are mandatory components in mostly all foods in Canada, and the presence of those elements was required for products to be collected in FLIP, all food labels in this sample displayed such information. However, quantitative ingredient declarations characterizing the amount of ingredients (e.g., percentage of fruit in a canned fruit product), was available for only 2.6% of products (*n* = 241/9379), and mostly in the following food categories: cereal and grain products (*n* = 71/241), beverages (*n* = 54/241), confectionery (*n* = 33/241) and bakery products (*n* = 27/241). Supplementary nutrition information was also only used in a small proportion of products (1.2%), of which only GDA symbols were identified in the analyzed food products; none of the products carried traffic light or health star rating symbols. Ninety-three percent (93%) of the total products with GDA labels were found in the following categories: bakery products (*n* = 50/114), cereal and related products (*n* = 41/114) and confectionary (*n* = 15/114). 

More than half of the foods and beverages in this sample (52%) carried some type of nutrition marketing, according to the INFORMAS Taxonomy (Table 2). Nutrient content claims were the most prevalent type of claims used on labels (46%), followed by health-related ingredient claims (17%). Such claims were primarily related to the presence of whole grains, fruits, vegetables or other plant-based ingredients (data not shown). Nutrient or other function claims were the least frequent, being displayed on only 0.8% of foods. Health claims were present on 7% of labels, and mostly consisted of reduction of disease risk claims and general health claims (4.1% and 2.7%, respectively). 

### 3.2. Proportion of Foods and Beverages in Each Tier

Overall, most food products were classified under Tier 2 and Tier 3 (Table 2). Foods classified under “Others” were mostly products “not in line with CFG” (98%, *n* = 1610/1613). 

**Figure 1:**
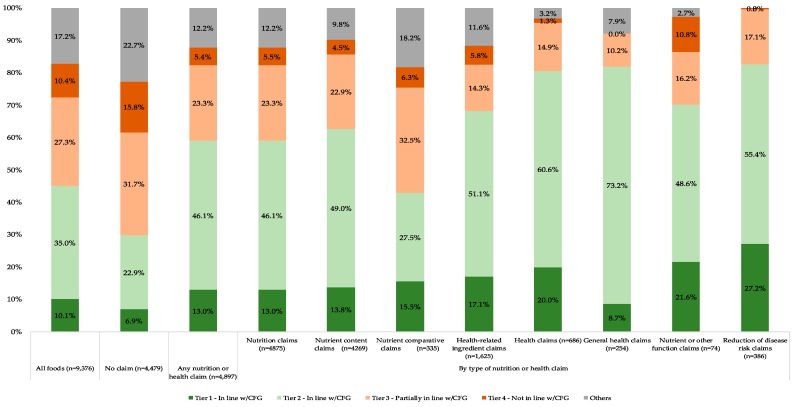
Proportion of foods and beverages in each tier by type of nutrition marketing ^1,2,3,4,5^. ^1^ Label components were classified using the INFORMAS step-wise approach proposed by Rayner and colleagues (adapted from: Rayner, M., et.al., Obes Rev, 2013. 14 Suppl 1: pp. 70–81) [44]. ^2^ The Health Canada Surveillance Tool (HCST) is a nutrient profiling system developed to assess Canadians’ adherence to Canada’s Food Guide in terms of amount and the quality of food choices [55]. ^3^ A label can include multiple classifications of nutrition and health claims therefore the total can be >100%. ^4^ Logos used by national heart foundations such as the British Heart Foundation or the Australia/New Zealand Heart Foundation Tick were considered as reduction of disease risk claims. In Canada, a similar symbol was used at the time of data collection (the Heart and Stroke Foundation logo) and was therefore classified as such for the purpose of this study; however, the logo did not comply with the Canadian regulations for disease risk reduction claims and it could instead be considered as an unregulated general health claim. The Heart and Stroke Foundation logo was discontinued in 2014. ^5^ Foods within each of CFG’s food groups were classified into four tiers, based on their fats, sugars and sodium content, or “Others”) as indicated in Table 1. HCST—Health Canada Surveillance Tool; QUID—Quantitative ingredient declaration; NFt—Nutrition Facts table; CFG—Canada’s Food Guide; Alt—Alternatives.

When the distribution of foods carrying nutrition marketing (*n* = 4897) within each tier was analyzed by the type of claim (Figure 1), it was found overall that foods carrying claims were primarily classified in Tier 1 and Tier 2 (59.1%), although over a quarter of these foods were also classified in Tier 3 and Tier 4 (23.3% and 5.4%, respectively). As expected, the distribution of foods into the Tiers varied across different types of claims. For example, products carrying health claims (>80%) were most likely classified as Tier 1 and Tier 2, although the overall prevalence of such products within the sample was limited (7.3%). 

Foods carrying nutrient comparative claims were primarily found in Tier 3, Tier 4 or “Others” (57%). Table 3 shows the logistic regression coefficients (unadjusted and adjusted model for food category) used to estimate the association of a product carrying each type of claim to being “in line with CFG” compared to those without claims that are also “in line with CFG”. Overall, in unadjusted models, the presence of almost all types of claims (except for nutrient comparative claims) significantly increased the likelihood that a product would be “in line with CFG”, as evidenced by their positive and significant coefficients (Table 3). 

For example, foods carrying nutrition claims or health claims are 21% and 73% more likely to be “in line with CFG” than products without those claims, respectively.

Nutrient comparative claims showed a negative value (i.e., a product is likely “not in line with CFG” if this claim is featured on the label), but it was not significant (*p* = 0.42). Products with nutrient comparative claims were further analyzed to determine possible reasons for the lack of association and it was found that 56% of foods with such claims fell within snacks (*n* = 51/335), meat and meat alternatives including mostly ham, salami, burgers, bacon (*n* = 66/335), ice creams and desserts (*n* = 58/335) and sugars and honey (*n* = 15/335); food categories highly discouraged in dietary guidelines. Such results are not surprising since, for example, all products within sugars and honey were classified as “Others” and 78% of snacks were classified as Tier 3 and Tier 4 (Table 2). When the adjusted models were analyzed, all types of nutrition and health claims were found to be significantly more likely to be “in line with CFG”. Such analyses suggest that not only the presence, but also the type of claim is associated with food category. 

## 4. Discussion

Packaged foods and beverages, which frequently display nutrition marketing, are commonly purchased by Canadians. This study investigates the alignment of a sample of Canadian packaged foods and beverages carrying nutrition marketing with the 2007 Canada’s Food Guide recommendations, using the Health Canada Surveillance Tool to provide baseline data upon the implementation of the new CFG guideline. In addition, this study also examined the use of other label components, such as supplementary nutrition information and QUID, which are currently not mandatory in Canada. 

With respect to the alignment of foods and beverages with the CFG’s recommendation, most products in this sample were considered Tier 2, Tier 3, and “Others” (predominantly those “not in line with CFG”, such as high calorie beverages, high-fat and/or sugar foods, and saturated and/or trans fats and oils [15]). Interestingly, these results mirror the findings from another study that investigated the nutritional quality of diets in Canadian individuals using the HSCT, which found most dietary choices made by Canadians were from Tier 2, Tier 3 as well as “Others not in line with CFG”. Our study therefore aligns with previous research that has suggested consuming packaged foods might restrict healthy eating [23,57]. 

Despite the NFt being reported as the primary source of nutrition information used by Canadians [58], consumers may not be able to easily identify and understand the nutrition information provided on the NFt [59] and therefore may rely on nutrition marketing that mostly appears on the front of packages to make purchasing decisions [60]. Results in the present study revealed that the presence of nutrition marketing increases the association of packaged foods of being aligned with CFG recommendations. This suggests that nutrition marketing may be a good indicator of healthier choices. However, consumers should be aware that many foods with nutrition marketing still fall outside CFG’s recommendations. For instance, 23.3% of foods with nutrition marketing in this study were classified as Tier 3 and 17.6% of foods with claims were classified into Tier 4 and “Others”. Moreover, 32% of foods with nutrient comparative claims were classified as Tier 3 and likely to be found in snacks, meat and meat alternatives, ice creams and desserts, and sugars and honey food categories. Moreover, since its inception, CFG has been criticized for being “obesogenic” [61,62] due its lack of cautionary advice to consumers about the consequences of consuming excess calories, as well as products from Tier 4 and “Others” (mostly high energy foods and beverages) [14,61]. This is concerning given that almost one-third of products consumed by Canadians come from foods in Tier 4 and “Others”, sources not recommended by the CFG [15]. While this study suggests nutrition marketing is associated with better alignment to the CFG, there are still many foods with nutrition marketing that are of poor nutritional quality, and therefore consumers should be cautious when using nutrition marketing for dietary decision making [63,64]. 

The use of other label elements, such as mandatory supplementary nutrition information (i.e., front-of-pack [FOP] labelling) could strengthen the nutrition information given to consumers and could help them to identify foods that exceed thresholds for nutrients of public health concern such as sodium, sugars and saturated fats [65]. Findings from the current study showed that the presence of supplementary nutrition information in Canada is low (1.2%) and was displayed primarily in the form of GDA. GDA have been identified in other research as the least effective system for consumers to differentiate “healthier” from “less healthy” food options [66,67,68]. Notably, a proposal to regulate the use of supplementary nutrition information in the form of “high in” symbols, a system that has been shown to be more effective [69,70] and identified as a key recommendation during CFG consultations [16], has recently been issued in Canada [65].

Although the prevalence of nutrition marketing on Canadian labels has been previously examined [25], benchmarking to international standards and comparisons to other studies were limited due to jurisdictional differences in nutrition marketing terminology. By coding claims using the standardized approach outlined by INFORMAS [44,48], comparisons are now feasible. This analysis showed that overall food labels in Canada are more heavily marketed (i.e., prevalence of nutrition marketing) (52%) than in New Zealand (39%) [51], Slovenia (39%) [29], the UK (32%) [52], five European countries (26%) [50], and Thailand (25%) [53], although Canada showed similar proportions to Australia (56%) [27]. However, in many of those countries other nutrition regulations are already in place to restrict the use of nutrition marketing on foods with an “unhealthy” nutritional profile; for example, through the use of nutrient profiling models to determine if a food is eligible to carry certain claims [71,72]. If such regulations were implemented in Canada, it may be even more likely that foods with nutrition marketing would align with dietary guidelines, and less likely to be on foods high in nutrients of public health concern. 

There are some limitations to this present analysis. First, a convenience sample was used instead of the full 2013 FLIP database. However, this sample included most foods that have been associated with increased risk of diet-related NCDs and “healthier” food patterns. Second, the data was collected five years ago and therefore, some products could have changed labels, could have been reformulated, or even been removed from the market. However, an assessment with respect to the 2007 CFG recommendations is valuable since such guideline was current at the time of data collection. In addition, the nutrient profiling model (i.e., HCST), developed based on the 2007 CFG, has not been updated with the revamp of the 2019 CFG and it is still the most up-to-date government-led nutrient profiling in Canada. It is also unknown whether the HCST will be updated to reflect new recommendations and when such upgrade could occur. Therefore, these results can provide baseline data upon the implementation of the 2019 CFG and be used to assess changes overtime once the new guideline is fully integrated to dietary practices. As such, studies assessing nutrition marketing and their association with new the guideline are encouraged. Third, although label components were classified using a standard approach, some misclassification could still have occurred, given the numerous styles and wording of claims found on food labels. When doubts arose, researchers discussed them and agreed upon a final classification. 

This study has a number of strengths. Firstly, at the time of collection, FLIP 2013 was the largest and most comprehensive branded food and beverage database in Canada, and included products sold nationally as well as private label brands. Secondly, the HCST has been validated for use on Canadian packaged foods [56]. Thirdly, the INFORMAS taxonomy was developed based on the CODEX Alimentarius international food standards; it therefore provides a global approach to classify label components, which has already been used in a number of studies worldwide [50,51,52,53,73]. As previously reported, many foods and beverages display regulated and unregulated nutrition and health claims on packages (identified as per Canadian regulations and guidelines) [25]. However, the use of an international standardized label taxonomy highlighted the routine use of health-related ingredient claims (e.g., “made with fruit”) to communicate nutrition information, which fall outside Canadian nutrition labelling regulations. In addition, Canada does not require the use of QUID in products carrying those claims, unlike many countries in Europe [74,75] and in Australia [76]. The latter also highlights lack of transparency to consumers regarding the contribution of certain ingredients in packaged foods. Moreover, other Canadian studies have evaluated “front-of-pack” symbols (both prevalence and nutritional quality) [63,77,78]; however, given that there is currently no government-led “front-of-pack” labelling regulations in Canada, many industry-led “front-of-pack” symbols highlight positive product attributes without emphasizing nutrients of public health concern. 

## 5. Conclusions

In conclusion, this study found that packaged foods with nutrition marketing are more likely to align with 2007 CFG’s recommendations than foods without nutrition marketing. However, consumers should be aware that nutrition marketing can also be found on foods highly discouraged by dietary guidelines. Using standardized methods to classify different label components can be useful to identify gaps in nutrition regulations (such as the lack of QUID and regulated supplementary nutrition information on Canadian labels) and can be used to compare results with other countries where more extensive policies and regulations have been introduced. This study can provide important baseline data to assess outcomes once the new CFG and other nutrition labelling policies in Canada are fully in place.

## Figures and Tables

**Table 1:** Health Canada Surveillance Tool Tier Thresholds ^1^.

Tier	Alignment to CFG	Conditions: Fats, Sugars and Sodium Content of Foods		Thresholds
**1**	Foods in line with CFG	Foods that do not exceed any of the three lower thresholds for total fat, sugars and sodium ^2^		**Lower thresholds:** Total Fat: ≤3 g/RA
**2**	Foods in line with CFG	Foods that exceed one or two lower thresholds for total fat, sugars or sodium, without exceeding any upper thresholds		Sugars: ≤6 g/RA Sodium: ≤140 mg/RA
**3**	Foods partially in line with CFG	**Vegetables and Fruit and Grain Products**	**Milk and Alternatives and Meat and Alternatives**	
		Foods that exceed all three lower thresholds without exceeding any upper thresholds for total fat, saturated fat, sugars or sodium, **or**	Foods that exceed all three lower thresholds without exceeding any upper thresholds for total fat, sugars or sodium ^3^, **or**	
		Foods that exceed only one upper threshold for total fat, saturated fat, sugars or sodium	Foods that exceed only one upper threshold for total fat, sugars or sodium ^3^, **or**	
			Foods that only exceed the upper saturated fat threshold	
**4**	Foods not in line with CFG	**Vegetables and Fruit and Grain Products**	**Milk and Alternatives and Meat and Alternatives**	**Upper thresholds:** Total Fat: >10 g/RA, Sugars: >19 g/RA, Sodium: >360 mg/RA,
		Foods that exceed at least two upper thresholds for total fat, saturated fat, sugars or sodium	Foods that exceed at least two upper thresholds for total fat, sugars or sodium ^3^	Saturated fat: >2 g/RA
“Others”	Foods in line with CFG	Water, unsaturated fats and oils ^4^		
	Foods not in line with CFG	High calorie beverages (≥40 kcal/100g), low calorie beverages (<40 kcal/100 g), high fat and/or sugar foods, meal replacements, saturated and/or trans fats and oils, supplements, ingredients/seasonings		

^1^ Adapted from Health Canada’s The Development and Use of a Surveillance Tool: The Classification of Foods in the Canadian Nutrient File According to Eating Well with Canada’s Food Guide, 2014 [56]. ^2^ Cannot exceed the upper threshold for saturated fat. ^3^ Irrespective of saturated fat content (value may be above or below upper saturated fat threshold). ^4^ HCST has defined unsaturated fats and oils as: “a fat or oil, (for example: salad dressing, margarine or mayonnaise) that has greater than or equal to 50% kilocalories from fat and 2 grams or less saturated and trans fatty acids combined per reference amount. If the fat or oil did not meet these criteria, it was classified in saturated and/or trans fats and oils”. CFG—Canada’s Food Guide, RA—Reference Amount.

**Table 2:** Proportions of label components and HCST Tier classification on a sample of Canadian packaged foods (*n* = 9376) ^1,2,3^.

	**Bakery (*n* = 2083)**		**Beverages (*n* = 1124)**		**Cereals (*n* = 1218)**		**Ice Creams & Desserts (*n* = 820)**		**Confectionary (*n* = 437)**		**Sugar & Honey (*n* = 193)**		**Snacks (*n* = 558)**	
	***n***	**%**	***n***	**%**	***n***	**%**	***n***	**%**	***n***	**%**	***n***	**%**	***n***	**%**
**Label Components**														
List of ingredients	2083	100%	1124	100%	1218	100%	820	100%	437	100%	193	100%	558	100%
Foods with QUID	27	1.3%	54	4.8%	71	5.8%	12	1.5%	33	8%	10	5.2%	18	3.2%
Nutrient declarations (NFt)	2083	100%	1124	100%	1218	100%	820	100%	437	100%	193	100%	558	100%
Supplementary Nutrition Information	50	2.4%	0	0.0%	41	3.4%	0	0.0%	15	3%	0	0.0%	6	1.1%
Any nutrition or health claim ^3^														
Yes	1153	55.4%	790	70.3%	675	55.4%	426	52.0%	150	34%	43	22.3%	405	72.6%
Nutrition claims	1146	55.0%	790	70.3%	672	55.2%	426	52.0%	146	33%	43	22.3%	405	72.6%
Nutrient content claims	970	46.6%	736	65.5%	614	50.4%	351	42.8%	79	18%	28	14.5%	378	67.7%
Nutrient comparative claims	41	2.0%	37	3.3%	11	0.9%	58	7.1%	3	1%	15	7.8%	51	9.1%
Health-related ingredient claims	518	24.9%	329	29.3%	347	28.5%	105	12.8%	75	17%	4	2.1%	112	20.1%
Health claims	215	10.3%	112	10.0%	155	12.7%	28	3.4%	5	1%	1	0.5%	21	3.8%
General health claims	149	7.2%	53	4.7%	23	1.9%	11	1.3%	5	1%	0	0.0%	2	0.4%
Nutrient or other function claims	17	0.8%	0	0.0%	10	0.8%	15	1.8%	0	0%	1	0.5%	0	0.0%
Reduction of disease risk claims ^4^	57	2.7%	72	6.4%	127	10.4%	2	0.2%	0	0%	0	0.0%	19	3.4%
No	930	44.6%	334	29.7%	543	44.6%	394	48.0%	287	66%	150	77.7%	153	27.4%
**HCST Tier Classification ^5^**														
Tier 1 - In line w/CFG	216	10.4%	40	3.6%	454	37.3%	33	4.0%					7	1.3%
Tier 2 - In line w/CFG	1103	53.0%	548	48.8%	583	47.9%	213	26.0%					113	20.3%
Tier 3 - Partially in line w/CFG	520	25.0%	54	4.8%	73	6.0%	445	54.3%					308	55.2%
Tier 4 - Not in line w/CFG	244	11.7%			18	1.5%	129	15.7%					130	23.3%
Others			482	42.9%	90	7.4%			437	100%	193	100%	-	-
	**Processed fish (*n* = 440)**		**Meat and Meat Alt. (*n* = 908)**		**Sauces & spreads (*n* = 50)**		**FVNL (*n* = 1489)**		**Eggs (*n* = 56)**		**Total Products (*n* = 9376)**			
	***n***	**%**	***n***	**%**	***n***	**%**	***n***	**%**	***n***	**%**	***n***	**%**		
**Label Components**														
List of ingredients	440	100%	908	100%	50	100%	1489	100%	56	100%	9376	100%		
Foods with QUID	4	0.9%	1	0.1%	2	4.0%	9	0.6%	0	0.0%	241	2.6%		
Nutrient declarations (NFt)	440	100%	908	100%	50	100%	1489	100%	56	100%	9376	100%		
Supplementary Nutrition Information	0	0.0%	2	0.2%	0	0.0%	0	0.0%	0	0.0%	114	1.2%		
Any nutrition or health claim ^3^														
Yes	217	49.3%	339	37.3%	12	24.0%	657	44.1%	30	53.6%	4897	52.2%		
Nutrition claims	217	49.3%	339	37.3%	12	24.0%	654	43.9%	27	48.2%	4875	52.0%		
Nutrient content claims	208	47.3%	291	32.0%	5	10.0%	582	39.1%	27	48.2%	4269	45.5%		
Nutrient comparative claims	1	0.2%	66	7.3%	0	0.0%	52	3.5%	0	0.0%	335	3.6%		
Health-related ingredient claims	8	1.8%	7	0.8%	7	14.0%	113	7.6%	0	0.0%	1625	17.3%		
Health claims	21	4.8%	12	1.3%	0	0.0%	93	6.2%	23	41.1%	686	7.3%		
General health claims	1	0.2%	2	0.2%	0	0.0%	8	0.5%	0	0.0%	254	2.7%		
Nutrient or other function claims	16	3.6%	5	0.6%	0	0.0%	8	0.5%	2	3.6%	74	0.8%		
Reduction of disease risk claims ^4^	5	1.1%	5	0.6%	0	0.0%	77	5.2%	22	39.3%	386	4.1%		
No	223	50.7%	569	62.7%	38	76.0%	832	55.9%	26	46.4%	4479	47.8%		
**HCST Tier Classification ^5^**														
Tier 1 - In line w/CFG	50	11.4%	2	0.2%			143	9.6%			945	10.1%		
Tier 2 - In line w/CFG	174	39.5%	133	14.6%			405	27.2%	11	19.6%	3283	35.0%		
Tier 3 - Partially in line w/CFG	141	32.0%	453	49.9%			524	35.2%	45	80.4%	2563	27.3%		
Tier 4 - Not in line w/CFG	75	17.0%	320	35.2%			56	3.8%			972	10.4%		
Others					50	100%	361	24.2%			1613	17.2%		

^1^ Label components were classified using the INFORMAS step-wise approach proposed by Rayner and colleagues (Adapted from: Rayner, M., et.al., Obes Rev, 2013. 14 Suppl 1: pp. 70–81) [44]. ^2^ The Health Canada Surveillance Tool (HCST) is a nutrient profiling system developed to assess Canadians’ adherence to Canada’s Food Guide in terms of amount and the quality of food choices [55]. ^3^ A label can include multiple classification of nutrition and health claims therefore total can be >100%. ^4^ Logos used by national heart foundations such as the British Heart Foundation or the Australia/New Zealand Heart Foundation Tick were considered as reduction of disease risk claims. In Canada, a similar symbol was used at the time of data collection (the Heart and Stroke Foundation logo) and was therefore classified as such for the purpose of this study; however, the logo did not comply with the Canadian regulations for disease risk reduction claims and it could instead be considered as an unregulated general health claim. The Heart and Stroke Foundation logo was discontinued in 2014.^5^ Foods within each of CFG’s food groups were classified into four tiers, based on their fats, sugars and sodium content, or “Others”) as indicated in Table 1. HCST—Health Canada Surveillance Tool; QUID—Quantitative ingredient declaration; NFt—Nutrition Facts table; CFG—Canada’s Food Guide; FVNL—Fruit, Vegetables, Nuts, Legumes; Alt—Alternatives.

**Table 3:** Associations of foods carrying different types of claims to CFG recommendations (*n* = 9376) ^1,2^.

	Total	In Line with CFG		Unadjusted Model			Adjusted Model		
	*n*	*n*	*%*	β-Coefficients	SE		β-Coefficients	SE	
Any nutrition or health claim									
Absent+	4479	1338	29.9%						
Present	4897	2893	59.1%	1.22	0.044	***	1.36	0.052	***
Nutrition claims									
Absent+	4501	1351	30.0%						
Present	4875	2880	59.1%	1.21	0.044	***	1.35	0.052	***
Nutrient content claims									
Absent+	5107	1551	30.4%						
Present	4269	2680	62.8%	1.35	0.044	***	1.50	0.053	***
Nutrient comparative claims									
Absent+	9041	4087	45.2%						
Present	335	144	43.0%	−0.09	0.112	ns	0.52	0.127	***
Health-related ingredient claims									
Absent+	7751	3122	40.3%						
Present	1625	1109	68.2%	1.16	0.058	***	0.91	0.068	***
Health claims									
Absent+	8690	3678	42.3%						
Present	686	553	80.6%	1.73	0.099	***	1.51	0.111	***
General health claims									
Absent+	9122	4023	44.1%						
Present	254	208	81.9%	1.75	0.164	***	1.36	0.178	***
Nutrient or other function claims									
Absent+	9302	4179	44.9%						
Present	74	52	70.3%	1.06	0.255	***	1.13	0.281	***
Reduction of disease risk claims									
Absent+	8990	3912	43.5%						
Present	386	319	82.6%	1.82	0.136	***	1.69	0.157	***

^1^ Main effects of binary logistic regression models (unadjusted and adjusted for food category). Foods classified in Tier 1, Tier 2 and water were considered “in line with Canada’s Food Guide“. ^2^ Logos used by national heart foundations such as the British Heart Foundation or the Australia/New Zealand Heart Foundation Tick were considered as reduction of disease risk claims. In Canada, a similar symbol was used at the time of data collection (the Heart and Stroke Foundation logo) and was therefore classified as such for the purpose of this study; however, the logo did not comply with the Canadian regulations for disease risk reduction claims and it could instead be considered as an unregulated general health claim. + Indicates reference category. The significance of the coefficients is indicated by the following codes: ns *p* > 0.05, *** *p* < 0.001. CFG—Canada’s Food Guide; SE—Standard Error.

## Supplementary Materials

The following are available online at http://www.mdpi.com/2072-6643/11/2/411/s1, Supplementary Table S1: Examples of health-related claims under the INFORMAS taxonomy Supplementary Table S2: Global Food Monitoring Group food categories.
