# Supplementary material for: Assessment of Packaged Foods and Beverages Carrying Nutrition Marketing against Canada’s Food Guide Recommendations
Source: Nutrients. 2019 Feb 15;11(2):411. doi: 10.3390/nu11020411 (PMC6412409; doi:10.3390/nu11020411)
Supplement: Supplementary file 1 [file nutrients-11-00411-s001.pdf]

Supplementary Table 1. Examples of health-related claims under the INFORMAS taxonomy[1]

**Types of health-related claims**			**Examples**
**Nutrition Claim**			
Nutrient content claim	Fibre	Sodium/Salt	"Source of protein"
	Energy	Cholesterol	
	Antioxidants/vitamins/minerals/hormones	Omega 3	
	Carbohydrates	Taurine/guanine	
	Fats	Caffeine	
	Sugar	Protein	
Nutrient comparative claim	Reduced fat	Reduced calories	"Reduced in sodium"
	More calcium	More fibre	
	Less sodium/salt	Reduced carbohydrates	
	Reduced sugar	More protein	
Health-related ingredient Claim	Wholegrain	Grains/seeds	"Contains wholegrain"
	Fruits/nuts/honey	Milk/cream	
	Vegetables/plants	Edible oils/oil emulsions	
	Bacteria/culture/probiotics/prebiotics	Cocoa/cacao	
**Health Claims**			
General health claim	General health claim	Specific body systems	"Healthy", "Maintaining healthy body weight"
	Super, healthy,	Digestive health	
	Low GI/energy density	Bones health	
Nutrient and other function claim	Nutrient + nutrient absorption	Nutrient + muscle	
	Nutrient + bone	Nutrient + digestion	"Probiotic cereal that contributes to healthy gut flora"
	Nutrient + growth	Nutrient + immunity	
	Nutrient + energy	Nutrient + overall health	
	Nutrient + strength	Nutrient + brain	
Reduction of disease risk Claim	Heart-related	Diabetes	"A healthy diet low in saturated and *trans* fats may reduce the risk of heart disease"
	Heart Foundation logos[2]	Glycaemic impact	
	Nutrient absorption	Osteoporosis	
	Cholesterol absorption		
**Other Claims**			
Other health-related claims	Other health-related claim		"Gluten-free"
	Environment claim		"Organic"

[1]Adapted from *Rayner, et al, Monitoring the health-related labelling of foods and non-alcoholic beverages in retail settings. Obes Rev, 2013. 14 Suppl 1: p. 70-81.* "A taxonomy of health-related food labelling for INFORMAS"; Rayner, M. and S. Vandevijvere. *INFORMAS Protocol: Food Labelling Module*. 2017. Available from: https://figshare.com/articles/INFORMAS_Protocol_Food_Labelling_Module/5673643 [2] Logos used by national heart foundations such as the British

Heart Foundation or the Australia/New Zealand Heart Foundation Tick were considered as reduction of disease risk claims. In Canada, a similar symbol was used at the time of data collection (the Heart and Stroke Foundation logo) and therefore classified as such for the propose of this study; however, it is important to note that the claim did not comply with the Canadian regulations for disease risk reduction claims and it could be considered instead as an unregulated general health claim. The Heart and Stroke Foundation logo was discontinued in 2014.

Supplementary Table 2. Global Food Monitoring Group food categories[1]

	Food category	Description
**Beverages**	Juices	Fresh and ambient fruit and vegetable juices
	Soft drinks	Sugar-sweetened and artificially-sweetened soft drinks
	Electrolyte drinks	Sports electrolyte drinks
	Waters	Plain and flavored waters
	Coffee and tea	All coffee and tea products
	Powdered beverages	All beverage mixes
**Bread and bakery products**	Bread	White, wholemeal and mixed grain/seed sliced bread and rolls
		Fruit bread and fruit-based muffins/rolls
		Wraps and other flatbread products
		Turkish pide, bagels, English-style muffins, crumpets, pizza bases and other plain bread-based products
	Biscuits and cookies	Filled and unfilled sweet biscuits
		Flavored and plain crisp bread and crackers
	Cakes, muffins and pastry	Scones, pikelets, doughnuts, cakes, sweet buns, pancakes, crepes, muffins (cake-style), slices etc
		Cake, pikelet and pancake dry mixes
		Sweet pastries (fresh, ambient, chilled and frozen)
**Cereal and grain products**	Breakfast cereal	Ready to eat breakfast cereals
		Oats and other breakfast cereals that require heating
		Other processed cereals (e.g. bran)
	Cereal and nut-based bars	Plain, chocolate-topped and yoghurt-topped cereal-based bars
	Noodles	Plain dry noodles
		Savory/flavored dry noodle-based dishes
	Pasta	Canned and ambient pasta and sauce (with and without meat) products (excludes frozen ready meals)
		Packaged fresh pasta with sauce
		Savory/flavored dry pasta-based side dishes
		Plain dry pasta
	Rice	Plain rice
		Savory rice-based side dishes
	Other breakfast products	All breakfast products not included in “Breakfast cereal”
	Other cereal products	Flour and other unprocessed cereals (e.g. polenta, cous cous, bread crumbs, yeast)
**Confectionery**	Chocolate and sweets	Chocolate-based confectionery, sugar-based confectionery
	Chewing gum	All sugar-sweetened and sugar-free chewing gums and bubble gum products

**Convenience foods**	Pizza	Frozen and refrigerated pre-packed pizzas
	Soup	Canned, chilled and ambient soup products
	Ready meals	Frozen, chilled and ambient pre-prepared meals
	Meal kits	Kits with ingredients to put meals together
	Pre-prepared salads and sandwiches	Chilled pre-prepared salads and sandwiches
	Other convenience foods	Other pre-prepared foods not included in above categories
**Dairy**	Cheese	Feta, haloumi, parmesan and other high-salt cheeses
		All types of full and reduced fat cheddar/Colby etc cheese including shredded, block or sliced
		Soft cheeses such as cream cheese, ricotta and cottage cheese
		Processed cheese slices and products
	Cream	Thickened, sour and regular cream products
	Desserts	Dairy-based desserts (e.g. custards, rice puddings)
		Dairy-based dessert mixes (e.g. powders)
	Ice cream and edible ices	Dairy and non-dairy-based ice cream varieties and edible ices
	Milk products	Flavored and unflavored dairy milk products
		Flavored and unflavored soymilks
		Flavored and unflavored oat, almond and other milks
		Condensed, evaporated and powdered milk products (including coconut milk)
	Yoghurt products	Fruit, flavored and natural yoghurts (full fat, reduced fat and skim varieties) including yoghurt drinks
**Edible oils**		Salted and unsalted butter and margarine products
**Fruit, vegetables, nuts and legumes**	Fruit	Dried fruit products including coconut
		Fruit-based bars
		Fruit products canned in juice or syrup
		Fruit gels, fruits in jelly and fruit puree
	Jam and fruit spreads	Jams, marmalades and other preserves
	Nuts and seeds	Salted and unsalted nuts and seeds
	Vegetables	Canned tomato products
		Canned beans and peas
		Baked beans in tomato sauce (with and without additions)
		Canned creamed, plain and sweet corn
		All other canned vegetables
		Pickled vegetable and olive products
		Frozen potato-based products
		Frozen vegetables

**Meat and meat alternatives**	Meat alternatives	Plain tofu and other meat-free alternatives
		Meat-free products (e.g. meat-free sausages)
	Processed meat	Pre-packed bacon products
		Beef, pork, chicken and lamb sausages and chilled hot dogs
		Pre-packaged sliced deli meats
		Pre-packaged salami and cured meats
		Beef, pork, chicken and lamb meat burgers
		Canned meat products (excluding soup and pasta)
		Frozen meat pies, sausage rolls and other meat-based pastry products
**Sauces, dressings and condiments**	Mayonnaise and salad dressings	Full and low-fat mayonnaise
		Oil-based, vinegar-based and other types of salad dressing
	Sauces	Table sauces such as tomato sauces and ketchups, sweet chilli, BBQ sauces
		Steak, HP and Worcestershire sauces
		Soy, fish, oyster and other Asian high-salt sauces
		Mustard products
		Marinade products
		Meat accompaniments (e.g. apple, cranberry and mint sauces)
		Plain and flavored tomato paste products
		Asian and Indian flavored powdered, ambient and liquid meal-based sauces
		Ambient and fresh pasta sauces
		Recipe bases
		Liquid and powdered gravies and stock
	Spreads	Crunchy and smooth salted and unsalted peanut butter
		Relishes, chutneys and pickles
		Other savory spreads (e.g. vegetable spreads)
		Pâté spreads
		Sweet spreads
		Yeast-extract spreads (e.g. vegemite)
		Chilled and ambient dips and salsa
**Eggs**		All egg products
**Fish and fish products**	Canned seafood	All varieties of plain and flavored canned seafood
	Chilled and frozen seafood	Chilled processed fish products (e.g. smoked salmon)
		Coated frozen fish products (e.g. fish fingers) and uncoated fish products

**Snack foods**		Plain and flavored potato crisps<br>Plain and flavored snack foods<br>Extruded snacks (e.g. cheesy snacks)<br>Plain and flavored corn chips<br>Pretzels, popcorn and other snackfoods<br>Other fried snackfoods (e.g. plantain chips)<br>All varieties of cracker-based snack packs
**Sugar, honey and related products**	Honey and syrups	Honey, golden, maple and other syrups<br>Dessert toppings<br>Sugar and artificial sweeteners

[1] Adapted from Dunford, E., *et al.* International collaborative project to compare and monitor the nutritional composition of processed foods. *Eur J Prev Cardiol* 19, 1326-1332 (2012); Rayner, M. and S. Vandevijvere. *INFORMAS Protocol: Food Labelling Module*. 2017. Available from: https://figshare.com/articles/INFORMAS_Protocol_Food_Labelling_Module/5673643
